# Inhibition of FicD-mediated AMPylation and deAMPylation by isoprenoid diphosphates

**DOI:** 10.1073/pnas.2533457123

**Published:** 2026-03-04

**Authors:** Aubrie M. Blevins, Wei Peng, Lisa N. Kinch, Zihan Monshad, Andrea G. Paredes, Christina Volz, Jared Rutter, Amanda K. Casey, Kevin G. Hicks, Kim Orth

**Affiliations:** ^a^Department of Molecular Biology, University of Texas Southwestern Medical Center, Dallas, TX 75390; ^b^Department of Biochemistry, University of Texas Southwestern Medical Center, Dallas, TX 75390; ^c^HHMI, Dallas, TX 75390; ^d^Department of Biochemistry, University of Utah, Salt Lake City, UT 84112; ^e^HHMI, Salt Lake City, UT 84112; ^f^Department of Nutrition and Integrative Physiology, University of Utah, Salt Lake City, UT 84112

**Keywords:** AMPylation, unfolded protein response, FicD, isoprenoid diphosphates, PTM

## Abstract

FicD regulates Unfolded Protein Response (UPR) through reversible AMPylation and deAMPylation of BiP, an HSP70 chaperone and master regulator of the UPR. FicD activity is regulated by endoplasmic reticulum-stress, catalyzing BiP AMPylation under low stress conditions to hold inactive chaperone in reserve. In stressed cells, FicD deAMPylates BiP, acutely increasing its active pool to assist in protein folding. Variants in UPR machinery, including those in the *FicD* gene, are linked to hereditary diseases. Despite the known role of FicD in UPR, in-vivo regulation of its activity remains elusive, and identifying metabolites that alter FicD activity could prove useful pharmaceutically. We applied an unbiased high-throughput screening platform, known as Mass spectrometry Integrated with equilibrium Dialysis for the discovery of Allostery Systematically (MIDAS), to identify small molecule metabolites that might regulate FicD activity. MIDAS revealed interactions between FicD and two mevalonate pathway intermediates: geranyl-pyrophosphate and farnesyl-pyrophosphate. Biochemical characterization indicates that both potently inhibit FicD-mediated AMPylation and deAMPylation. The crystal structure of FicD bound to farnesyl-pyrophosphate demonstrates a competitive inhibition mechanism, with the pyrophosphate adopting the alpha and beta phosphate positions of adenosine triphosphate (ATP) and the hydrocarbon chain filling the nucleoside pocket. FicD variants previously appeared as biochemically indistinguishable, yet lead to different human pathologies. We demonstrate farnesyl-pyrophosphate inhibits FicD^R374H^ and FicD^R374C^ variants implicated in causing hereditary spastic paraplegia, but not the FicD^R371S^ variant associated with neonatal diabetes. This study furthers our understanding of FicD inhibitors and distinguishes disease causing variants, providing insight into pharmacological targeting of UPR activity.

## Significance

FicD regulates Unfolded Protein Response (UPR) signaling in metazoans by fine-tuning BiP chaperone capacity. Therefore, targeting FicD activity may be a tractable method of altering UPR signaling for therapeutic benefit. We identify geranyl- and farnesyl-pyrophosphate as specific FicD inhibitors. Notably, these small molecules differentially inhibit disease-causing variants of FicD. A structure of farnesyl-pyrophosphate bound to the FicD active site helps explain the differential inhibition of pathogenic variants and provides insight into interactions that can be differentially exploited for modifying FicD activity. Their composition provides a chemical foundation for future drug development efforts targeting FicD activity.

The endoplasmic reticulum (ER) is a major hub of protein folding, assembly, modification, and transport. ER protein folding machinery is continuously challenged by dynamic fluctuations in ER protein load, and cells have evolved signaling mechanisms to sense and appropriately respond to stressors of this process (1–4). The Unfolded Protein Response (UPR) is an adaptive signaling pathway that both senses and responds to disruptions in ER protein homeostasis, or proteostasis (1, 4–7). When unfolded protein clients accumulate in the ER, UPR signaling reestablishes proteostasis through multiple mechanisms, including enhancing ER protein folding capacity and reducing the number of ER protein clients. The essential HSP70 chaperone BiP (HSPA5) serves as the master regulator of UPR activation through reversible association and inhibition of the UPR transducers IRE1, ATF6, and Protein kinase R-like endoplasmic reticulum (ER) kinase (PERK) (7–12). Maladaptive UPR activation is involved in a wide range of disease processes, such as cancer, neurodegenerative disease, and metabolic disease (3, 4, 6, 13–15). Significant efforts have focused on targeting the UPR and ER proteostasis for therapeutic development (3, 6, 14–16).

In metazoans, the ER transmembrane protein FicD acts as a rheostat for BiP chaperone activity via posttranslational AMPylation and deAMPylation (17–20). FicD is a bifunctional enzyme, catalyzing both AMPylation and deAMPylation of BiP in an ER stress-dependent manner. During homeostasis or low ER Stress, FicD acts as an AMPylator, generating a pool of inactive BiP (17, 19, 21). FicD-mediated AMPylation of the residue T518 in BiP’s substrate binding domain impairs J-protein stimulated ATP hydrolysis, locking BiP in its ATP-bound, chaperone-incompetent conformation (22). ER stress induces a shift in FicD activity from AMPylation to deAMPylation of BiP, thereby restoring its chaperone function and acutely increasing the pool of active BiP (21, 23, 24). This ER stress-dependent activity in FicD has been demonstrated in many cell culture models and animal models where FicD is especially important in terminally differentiated cells that experience repeated physiological stress (12, 17, 19, 21).

**Fig. 1.**
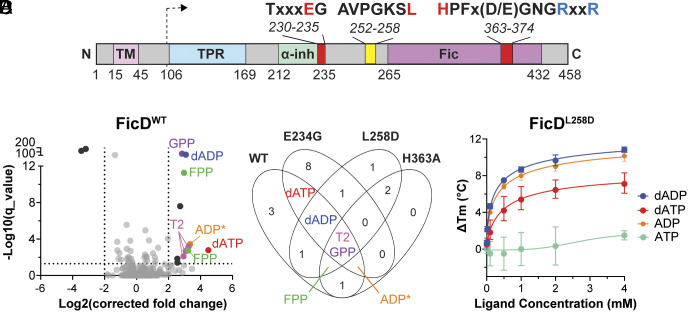
High-throughput FicD metabolite screen identified canonical ligands. (*A*) Domain diagram of FicD with labeled motifs that include functional (red) and pathogenic (blue) positions and the dotted arrow indicating the start site for expression constructs. (*B*) Volcano plot of FicD^WT^ MIDAS screen. Dotted lines indicate significance cutoffs (*Q* < 0.05 and |Log_2_ corrected fold change)| > 2). Significant PMIs are labeled and colored: GPP (purple), FPP (green), dADP (blue), dATP (red), ADP* for 3’,5’-ADP, ADP, or dGDP (orange), T2 (pink). Other PMIs are colored: significant but in <3 FicD construct screens (black) and nonsignificant (gray). (*C*) Venn diagram comparing significant PMIs detected in screens with WT, E234G, L258D, H363A. PMIs that overlap in 3 or more construct screens are labeled and colored as in *B* with nucleotides: ADP (orange), ATP (teal), dADP (blue), and dATP (red). (*D*) DSF melt point analysis of FicD^L258D^ with the indicated nucleotide (legend) in the presence of 5 mM MgCl_2._ Error bars represent three independent experiments.

The dual catalytic activity of FicD is mediated by a single Fido (Fic, Doc, and AvrB) domain with a conserved α-helical fold (20, 25, 26). Although other substrates can be used by Fido domains, the majority of Fido domain-containing enzymes, including FicD, use ATP and Mg^2+^ to catalyze AMPylation using a conserved motif: HPFx(D/E)GNGR_1_xxR_2_ (20, 21, 25–27) (Fig. 1*A*). A second conserved motif [S/TxxxE(G/N)] comprises an autoinhibitory helix (*α-inh*) in the FicD enzyme. The *α-inh* glutamate sidechain (E234 in human FicD) competes for the binding of the g-phosphate of ATP with the sidechain of conserved arginine (R374 in human FicD) in the catalytic motif (Fig. 1*A*). A salt bridge formed between E234 and R374 shifts FicD activity to deAMPylation (26, 28). Mutation of this glutamate to glycine (FicD^E234G^) breaks this salt bridge and abolishes this intramolecular autoinhibition, thereby, making FicD a constitutive AMPylator and abolishing deAMPylation activity (26, 29). The lack of regulation by *α-inh* is similar to the activity mediated by many bacterial pathogens that encode Fido-mediated activity, including VopS and AvrB (25, 30).

The oligomeric state of the metazoan FicD enzyme also regulates the AMPylation/deAMPylation activity. The enzyme forms a dimer in solution, and mutations in the dimer interface (FicD^L258D^) yield a monomeric enzyme (Fig. 1*A*). The monomerization of FicD results in increased flexibility of *α-inh,* which facilitates both AMPylation competent binding of ATP and increases in AMPylation activity relative to wild-type FicD (24, 31). However, the FicD^L258D^ mutant, in contrast to the constitutive AMPylator FicD^E234G^, retains the ability to deAMPylate (24, 31). Collectively, these studies have revealed the structural relevance of the *α-inh* regulatory salt bridge and dimerization of FicD.

The importance of the inherent switching activity from a deAMPylator to an AMPylator is further realized when studying virulence factors and disease variants of metazoan FicD. For example, in some bacterial Fido enzymes, the *α-inh* and the Fido domain encompass two separate proteins and are expressed as a toxin–antitoxin module (27). AMPylation activity is regulated by the ratio of toxin to antitoxin. In a *Neisseria* Fido enzyme, autoAMPylation relieves this intramolecular autoinhibition, facilitating AMPylation activity (32). In **Enterococcus faecalis*,* the Fido enzyme is regulated by levels of Mg^2+^ and Ca^2+^ to control its AMPylation/deAMPylation activity (33). In vitro, Ca^2+^ appears to inhibit the human FicD deAMPylation of BiP, but the physiological relevance remains unclear (33).

Studies in model organisms have illuminated both tissue and stress-dependent roles of FicD activity. In *Drosophila*, FicD (dFic) is required to maintain visual transmission in the eye (34). Flies deleted for *dFic* sustain increased eye damage and an impaired ability to recover from repetitive light stress (21) and locomotor impairment (35). In *FicD*^−/−^ mice, a pharmaceutical induction of UPR stress caused increased fibrosis in the pancreas (21) and reduced ER stress adaptation in the liver (36). Conversely, enhanced UPR stress in the absence of FicD protected against hypertrophy‐induced heart failure in mice (37). Additionally, *C. elegans* lacking *fic-1* show enhanced UPR induction and chaperone expression that limited polyglutamine toxicity (38). These studies suggest that targeting FicD activity in specific tissues may provide a beneficial strategy by altering UPR signaling.

Missense mutations in the FicD active site are linked to pathologies in human patients. A rare homozygous recessive FicD^R371S^ variant is associated with neonatal diabetes and severe neurodevelopmental impairment (39). A preclinical mouse model of this disease indicates FicD^R371S^ dysregulation causes progressive loss of islet organization and impaired insulin secretion (40). Two additional rare homozygous FicD variants, FicD^R374C^ and FicD^R374H^, are implicated in hereditary spastic paraplegia and a risk of diabetes in adulthood (41, 42). Notably, although the recessive FicD variants have similar catalytic activity with weakened AMPylation and no deAMPylation activity, they cause different pathologies. Interestingly, in both flies and mice, the FicD^E234G^ allele that encodes strong, constitutive AMPylation activity is a homozygous lethal variant (24, 43). The accumulation of AMPylated BiP caused by a loss of FicD deAMPylation activity in FicD^R371S^, FicD^R374H^, and FicD^R374C^ patients is likely central to disease progression, which highlights the possible benefits of targeting pathologic FicD activity.

In this work, we utilize an unbiased, high-throughput screen known as Mass spectrometry Integrated with equilibrium Dialysis for the discovery of Allostery Systematically (MIDAS), to investigate the possibility of small molecule metabolite regulation of FicD activity (44). Our screen identified the isoprenoid diphosphates geranyl-pyrophosphate (GPP) and farnesyl-pyrophosphate (FPP) as potent inhibitors of FicD-mediated AMPylation and deAMPylation. Orthogonal binding assays and in vitro activity assays reveal that the interactions between GPP/FPP and FicD are specific to the metazoan enzymes, as GPP/FPP is unable to inhibit the activities of bacterial proteins with Fido domains. A crystal structure of metazoan FicD bound to FPP reveals that FPP binds within the FicD active site in a manner analogous to ADP binding. Finally, we generated recombinant proteins for the human FicD variants known to cause human disease and observed FPP binding and inhibition for FicD^R374H^ and FicD^R374C^ variants, but not for the FicD^R371S^ variant. Overall, our work demonstrates regulation of the Fido domain by a class of small molecules, which may provide a scaffold for the development of specific FicD inhibitors.

## Results

### MIDAS Screen Reveals Putative Small-Molecule Metabolites That Interact with FicD.

To identify small-molecule metabolite interactions with FicD, we applied an unbiased high-throughput screening approach known as MIDAS (44). Given the high concentrations of protein required for MIDAS screening, we purified the soluble ER luminal domain (residues 105 to 458) of wild-type human FicD and three mutants FicD^E234G^, FicD^L258D^, and FicD^H363A^ for screening (Fig. 1*A*). These mutants were selected based on their well-established biochemical features (23, 24, 31). All three are predicted to enhance detection of protein–metabolite interactions (PMIs) relative to FicD^WT^ through increased affinity for nucleotide substrates (FicD^E234G^), improved solubility (FicD^L258D^), and loss of catalytic turnover (FicD^H363A^) (Fig. 1*A*). To decrease the potential for nonspecific small-molecule binding, we ensured the purity of our FicD proteins (*SI Appendix*, Fig. S1 *A* and *B*). As reported by other investigators (24, 31), we observe an increase in elution volume of the FicD^L258D^ mutant, consistent with its monomerization (*SI Appendix*, Fig. S1*C*). Finally, intact mass of screened proteins did not reveal obvious contamination (*SI Appendix*, Fig. S1*D*).

Out of the 602 naturally occurring human metabolites in the MIDAS library, we applied a significance cutoff (q value < 0.05) to identify 12 PMIs with a Log_2_ (corrected fold change) of >2 and two PMIs with a Log_2_ (corrected fold change) <-2 in the FicD^WT^ screen (Fig. 1*B* and *SI Appendix*, Table S2). Hits were additionally triaged based on detection of multiple adducts of the metabolite in a single dataset (FPP and T2 in Fig. 1*B*) and identification of the same metabolites across three or more of the FicD construct screens (Fig. 1*C*). Three chemical classes passed this initial triage: adenosine nucleotides, isoprenoid diphosphates, and thyroid hormone intermediates (Fig. 1 *B* and *C* and *SI Appendix*, Tables S2–S5). These hits were all significantly enriched, indicating a stable binding that did not result in catalytic turnover.

Two of the chemical classes that passed initial triage represent known FicD PMIs: adenosine nucleotides and thyroid hormone intermediates. FicD uses ATP and Mg^2+^ to catalyze AMPylation. ADP readily binds to FicD, whereas intramolecular autoinhibition of AMPylation activity destabilizes ATP binding (26, 28, 39). In agreement with the canonical activity of FicD, an adduct consistent with ADP and the closely related analog, deoxyadenosine diphosphate (dADP), were significantly enriched in three of the four FicD constructs. The adduct consistent with ADP was identified in FicD^WT^, FicD^E234G^, and FicD^H363A^ screens, while dADP was identified in FicD^WT^, FicD^E234G^, and FicD^L258D^ screens (Fig. 1 *B* and *C* and *SI Appendix*, Tables S2–S5). The FicD substrate ATP, although it did not pass the significance cutoff, was only detected in the screen with FicD^E234G^, which is not intrinsically autoinhibited (Dataset S2). However, the dATP analog was significantly enriched in the FicD^WT^ and FicD^E234G^ datasets (Fig. 1*C* and *SI Appendix*, Tables S2 and S3). dATP was also enriched in the FicD^L258D^ dataset, although it did not pass the significance cutoff (Dataset S3). Finally, previous drug screens of FicD have reported liothyronine, a thyroid hormone intermediate, as a small-molecule inhibitor of FicD (45). Consistent with these findings, 3,3’-Diiodothyronine (labeled T2 in Fig. 1*B*) was significantly enriched in all FicD mutant datasets (Fig. 1*C* and *SI Appendix*, Tables S2–S5). Overall, the detection of canonical FicD ligands served as internal positive controls for MIDAS screening, which supported our confidence in the previously unreported interactions discussed below.

A few spurious hits were enriched or depleted in only one FicD construct dataset (Fig. 1*B* and *SI Appendix*, Tables S2–S5). For example, the small, acidic compound glyoxylic acid was significantly depleted in FicD^WT^ (*SI Appendix*, Table S2), but not in the FicD mutant datasets (*SI Appendix*, Tables S3–S5). In MIDAS, depletion may indicate either a catalytic turnover that is faster than the rate of diffusion across the dialysis membrane, a noncovalent interaction that resists release from the interacting protein during methanol precipitation, or a covalent modification of the protein. Accordingly, glyoxylic acid is known to spontaneously and nonenzymatically react with protein sidechains, a modification known as glycation (46).

### MIDAS Identifies Analogues of Canonical FicD Ligands.

MIDAS screening identified putative interactions between FicD and dADP/dATP, which are the deoxy form of canonical FicD ligands ADP and ATP (Fig. 1 *B* and *C* and *SI Appendix*, Tables S2–S5). To validate the interaction between FicD and dADP/dATP, we performed differential scanning fluorimetry (DSF) to measure alterations in FicD’s thermal stability upon ligand binding. The monomeric FicD^L258D^ mutant produced a prototypical sigmoidal melt curve reflecting the melting temperature (T_m_) of a single unfolding transition state (*SI Appendix*, Fig. S2 *A* and *B*). Consistent with the MIDAS screen, addition of increasing dADP (*SI Appendix*, Fig. S2*A*) or dATP (*SI Appendix*, Fig. S2*B*) ligand concentrations lead to higher T_m_ values, indicating stabilization of the protein and binding of the metabolites.

The dose–response curves for FicD^L258D^ in the presence of dADP and dATP reached maximal shifts (~11 °C and ~7 °C, respectively) at saturating ligand concentrations, with EC50s of 0.28 mM and 0.53 mM, respectively (Fig. 1*D*). The dose–response curve for dADP mirrored that of ADP (~10 °C shift with EC50 of 0.37 mM, Fig. 1*D*), indicating a similar binding mode for the two ligands. Consistent with previous reports (26, 31), the binding curve for ATP did not show saturation at the indicated concentrations (Fig. 1*D*). The weak binding of ATP to FicDL258D is consistent with previous observations that *α-inh* flexibility conferred by the L258D mutation alters the ATP binding mode without altering ATP affinity (31). In contrast to ATP, the stable binding of dATP to FicDL258D suggests *α-inh* helix can accommodate the ligand with an increased affinity (Fig. 1*D*).

Because monomerization-induced flexibility of the FicD^L258D^ *α-inh* helix increases its ability to accommodate nucleotide ligands (26, 31), we used DSF to verify nucleotide binding to FicD^WT^. While the monomeric FicD^L258D^ displayed a monophasic melt transition with a T_m_ of 49.5 °C (*SI Appendix*, Fig. S2 *A* and *B*), the melt transition for dimeric FicD^WT^ was more complex, with a ~12 °C shift toward higher temperature (*SI Appendix*, Fig. S2 *C* and *D*). This T_m_ difference is consistent with previous reports comparing the monomeric and dimeric enzymes (31). The dose–response of ADP, dADP, and dATP for the FicD^WT^ (*SI Appendix*, Fig. S2 *A*–*E*) follow a similar trend to those for FicD^L258D^, with the loss of the 2’ hydroxyl in ribose trending toward slight improvements in binding. The minor differences in nucleotide affinity are consistent with previous reports, which note that monomerization does not alter ATP binding affinity, but rather it involves the positioning of ATP in the FicD active site (31). Given that FicD^L258D^ retains bifunctional AMPylation and deAMPylation activities with only modest differences in nucleotide binding, we leveraged the monophasic melt transition of the L258D mutant to facilitate dose–response analysis.

### Geranyl-Pyrophosphate and Farnesyl-Pyrophosphate Are Specific FicD Inhibitors.

**Fig. 2.**
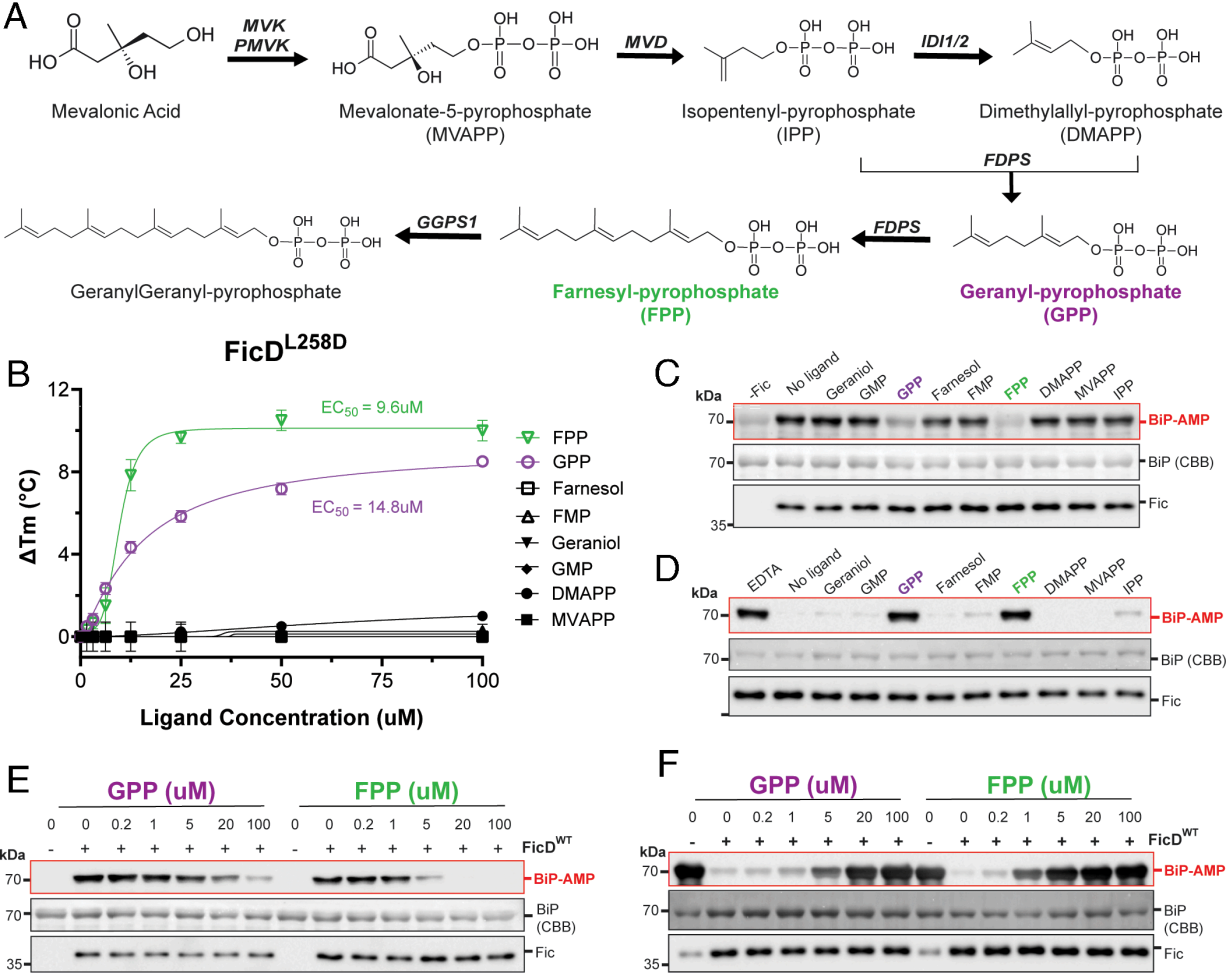
Geranyl-pyrophosphate and Farnesyl-pyrophosphate are specific FicD inhibitors. (*A*) Mevalonate Pathway for GPP and FPP biosynthesis in humans. Structures were created in MarvinSketch. (*B*) DSF melting of FicD^L258D^ with the indicated metabolites (legend) in the presence of 5 mM MgCl_2._ Error bars represent two or three independent experiments. (*C* and *D*) Western blot of (*C*) Δ26hBiP^T229A^ AMPylation and (*D*) deAMPylation of Δ26hBiP^T229A^ by FicD^WT^ in the presence of metabolites (100 μM) or EDTA (5 mM). (*E* and *F*) Western blots of GPP (*Left*) or FPP (*Right*) dose–response inhibition of (*E*) Δ26hBiP^T229A^ AMPylation and (*F*) deAMPylation Δ26hBiP^T229A^ by FicD^WT^. AMPylated BiP and FicD were quantified by immunodetection and total BiP was quantified by CBB in all activity assays.

MIDAS screening identified the isoprenoid diphosphate GPP to be significantly enriched in all four FicD mutant datasets, and the related metabolite FPP was significantly enriched in FicD^WT^, FicD^L258D^, and FicD^H363A^ (Fig. 1 *B* and *C* and *SI Appendix*, Tables S2–S5). Further support for these PMIs is reflected by multiple adducts of GPP and FPP being significantly enriched. GPP and FPP are pyrophosphate esters of the terpenoids geraniol and farnesol (47). GPP contains two isoprene units and FPP contains three isoprene units (Fig. 2*A*). In eukaryotes, they are intermediates of the mevalonate pathway, which produces isoprenoids, sterols, and lipids (Fig. 2*A*) (47, 48). In DSF, GPP and FPP elicited a dose-dependent and saturating increase in the DT_m_ of FicD^L258D^, yielding EC50s of 14.8 μM and 9.6 μM, respectively (Fig. 2*B*). Notably, these apparent binding parameters are ~20 to 30 fold tighter than that of ADP.

To ensure specificity of binding to the FicD enzyme, we performed DSF with Glutathione S-transferase (GST) and BiP in the presence of the same increasing metabolite concentrations. Neither GPP nor FPP elicited a change in the GST melt curves (*SI Appendix*, Fig. S3 *A* and *C*) or T_m_ (*SI Appendix*, Fig. S3 *B* and *D*) at the same concentrations measured for FicD. Like FicD, BiP is an ATP/Mg^2+^ dependent protein that could potentially leverage the same binding mode as GPP/FPP. However, neither GPP nor FPP elicited a change in the melt curves (*SI Appendix*, Fig. S4 *A* and *C*) or T_m_ of BiP (*SI Appendix*, Fig. S4 *B* and *D*). Together, these results indicate that binding of GPP/FPP at the evaluated concentrations is specific for the FicD protein.

To probe the structural specificity of the GPP/FPP metabolites, we tested binding of various analogs to FicD^L258D^ using DSF. The farnesol and geraniol isoprenoid chains lacking pyrophosphate did not bind to FicD^L258D^, nor did the monophosphate versions: geranyl-monophosphate (GMP) and farnesyl-monophosphate (FMP) (Fig. 2*B*). These results demonstrate the importance of the diphosphate in conferring binding to FicD. Similarly, dimethylallyl-pyrophosphate (DMAPP) composed of a single isoprene unit with a pyrophosphate did not bind to FicD^L258D^. Moreover, mevalonate-pyrophosphate (MVAPP), which is the precursor to DMAPP, also did not bind to FicD (Fig. 2*B*). Together, these binding studies indicate that the specific combination of at least two isoprene units and a diphosphate are required for FicD binding.

We next sought to assess the effects of FPP, GPP, and isoprenoid analogues on FicD activity. To accurately judge the effects of small molecules on FicD AMPylation activity, we used the ATPase defective BiP^T229A^ mutant as substrate (22, 49, 50). Loss of ATP hydrolysis by the BiP mutant ensures: 1. conformational homogeneity of the BiP protein substrate; 2. lack of BiP competition for the ATP substrate; and 3. no production of ADP, a competitive inhibitor for ATP. In addition, we deleted the first 25 amino acids of BiP for ease with its purification and solubility. While GPP and FPP at 100 μM concentration inhibited the AMPylation of Δ26hBiP^T229A^ by FicD^WT^, the analogs geraniol, farnesol, GMP, FMP, DMAPP, and MVAPP did not (Fig. 2*C*). Similarly, GPP and FPP at 100 μM concentration inhibited the deAMPylation activity of FicD^WT^ (Fig. 2*D*). However, geraniol, farnesol, GMP, FMP, DMAPP, and MVAPP did not affect FicD^WT^ deAMPylation activity (Fig. 2*D*). These results corroborate the findings of our DSF experiments above.

We then assayed the potency of GPP/FPP induced inhibition of AMPylation and deAMPylation activity. In vitro AMPylation and deAMPylation assays revealed dose-dependent inhibition of both AMPylation and deAMPylation activities of FicD (Fig. 2 *E* and *F*). Consistent with our DSF data, FPP was a more potent inhibitor of both AMPylation and deAMPylation activity than GPP. FPP inhibition of AMPylation and deAMPylation started as low as 5 μM and 1 μM, respectively (Fig. 2 *E* and *F*), while GPP inhibition of AMPylation and deAMPylation activity started as low as 20 μM and 5 μM, respectively (Fig. 2 *E* and *F*).

Finally, we explored the inhibitory effects of GPP/FPP on other Fido proteins to see if these metabolites could serve as generic inhibitors of Fido domains. To test this specificity, we performed in vitro activity assays with GPP/FPP and two constitutively active Fido proteins from bacteria: VopS from **Vibrio* parahaemolyticus* (25) and AvrB from *Pseudomonas syringae* (30). Neither GPP nor FPP affected the AMPylation activity of VopS or the rhamnosylation activity of AvrB (*SI Appendix*, Fig. S5).

**Fig. 3.**
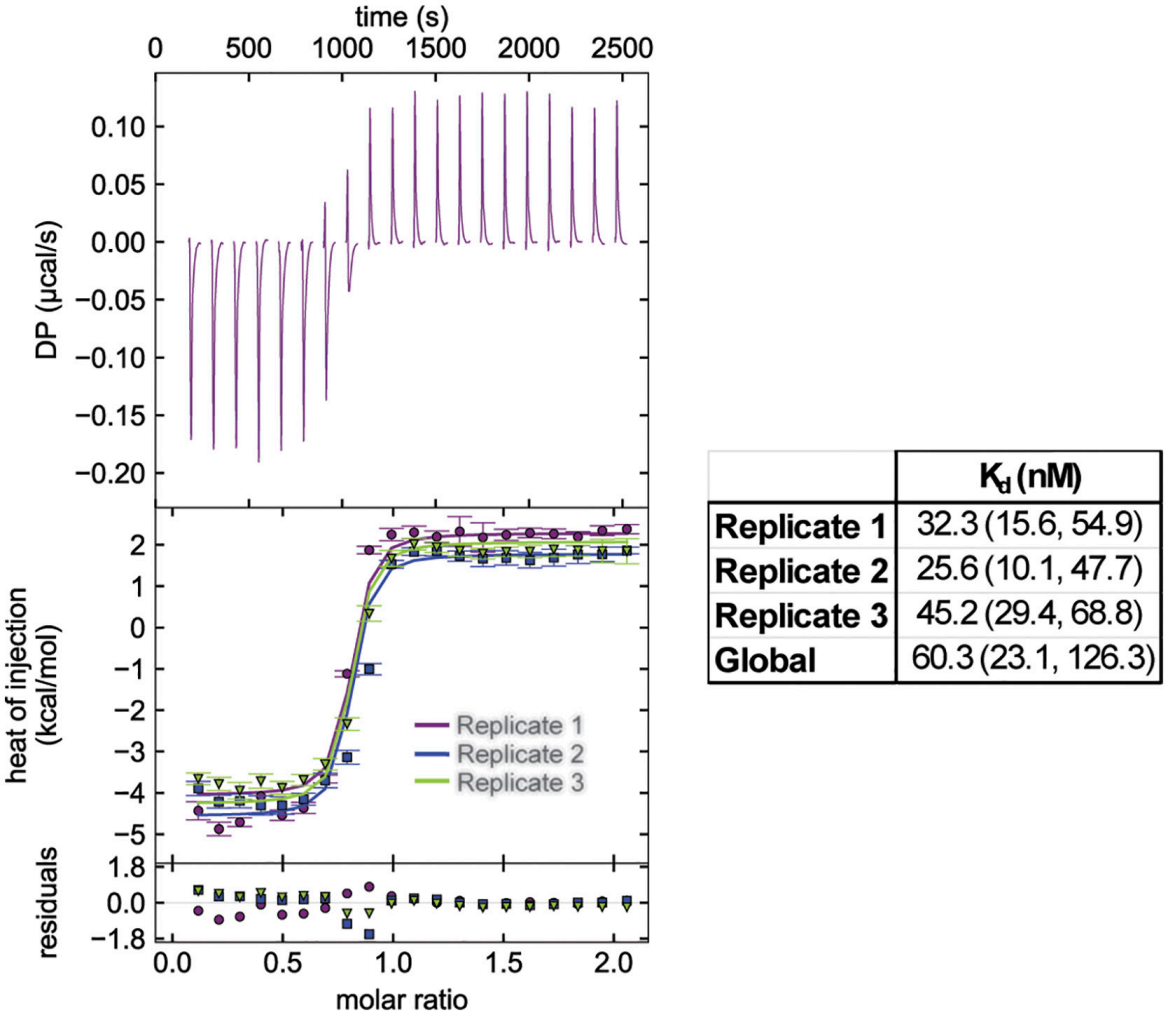
ITC with FicD and Farnesyl-pyrophosphate. Representative ITC thermogram for FPP binding to Δ104hFicD^L258D^ (*Upper*). Heats of injection of all three replicates are displayed (*Lower*). Dissociation constants (K_d_) of individual replicates and global fitting of triplicate isotherms are reported with 1σ error intervals in parenthesis.

DSF measurements suggested high-affinity binding of FPP and GPP to FicD when compared to nucleotides (Figs. 1*D* and 2*B*). To further support this notion, we used Isothermal Titration Calorimetry (ITC) to determine the K_d_ of FPP binding. We observed FicD^L258D^ bound to FPP with a K_d_ of approximately 60 nM at a MgCl_2_ concentration of 4 mM (Fig. 3). In these experiments, the interaction between FPP and Mg^2+^ generated endothermic peaks that interfered with detecting the interaction between FPP, Mg^2+^, and FicD (Fig. 3, *Upper*). The stoichiometry of FPP binding to FicD was consistently <0.85, which may indicate that Mg^2+^ was not saturating. Because the endothermic interaction between Mg^2+^ and FPP limited the quantity of MgCl_2_ we could add, the measured K_d_ could be larger than the true value. Nevertheless, the K_d_ of FPP binding was observed to be over one order of magnitude smaller than that for ADP, which is reported to be 1.5 μM for FicD^WT^ (26).

### Isoprenoid-Pyrophosphate Binding to FicD Requires Mg^2+^.

**Fig. 4.**
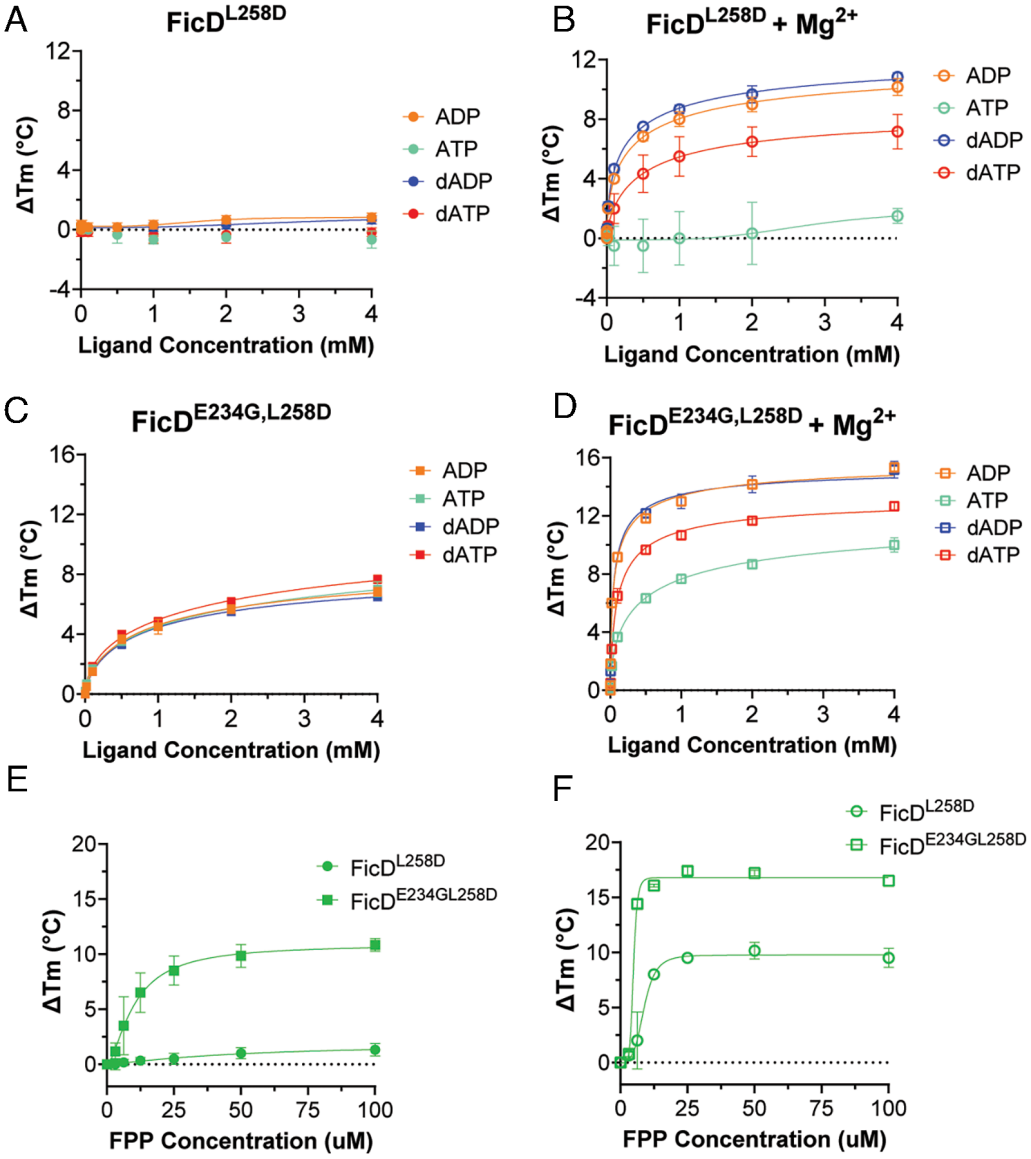
Mg^2+^ increases nucleotide and FPP binding to FicD^L258D^ and FicD^E234G, L258D^. (*A*–*D*) Dose–response FicD melting by DSF with nucleotides: ADP (orange), ATP (teal), dADP (blue), and dATP (red). (*A*) FicD^L258D^ in the absence or (*B*) presence of 5 mM MgCl_2_ (data displayed from 1D again for clarity) and (*C*) FicD^E234G, L258D^ in the absence (*D*) or presence of 5 mM MgCl_2_. (*E* and *F*) FPP-induced DSF melting of Fic^L258D^ and FicD^E234G, L258D^ (*E*) in the absence or (*F*) presence of 5 mM MgCl_2_. Error bars for all curves represent three independent experiments.

Our biochemical data indicate that FPP is a potent and specific inhibitor of FicD, whose binding and inhibition requires the pyrophosphate moiety. Given this requirement, we considered if the inhibitor pyrophosphate bound in the active site of the enzyme in a manner analogous to that of the nucleotide, which coordinates the α- and β- phosphates together with Mg^2+^ (26). Accordingly in DSF, the ADP, dADP, ATP, and dATP nucleotides did not bind to FicD^L258D^ in the absence of MgCl_2_ (Fig. 4*A*). Conversely, the addition of 5 mM MgCl_2_ conferred ADP, dADP, and dATP binding to FicD^L258D^ (Fig. 4*B*).

Interestingly in an alternate FicD variant, FicD^E234G, L258D^, all nucleotides were able to bind in the absence of MgCl_2_ (Fig. 4*C*). The addition of 5 mM MgCl_2_ further enhanced binding of these nucleotides (Fig. 4*D*). To assess if the pyrophosphate of FPP could coordinate Mg^2+^ in a manner analogous to that of the nucleotides, we tested if FPP binding also required Mg^2+^. Interestingly, the pattern of FPP binding to the FicD variants was like that of ADP, dADP, and dATP. In the absence of MgCl_2_, FPP did not bind to FicD^L258D^; whereas it bound to FicD^E234G, L258D^ (Fig. 4*E*). In the presence of 5 mM MgCl_2_, FPP bound to both FicD^L258D^ and FicD^E234G, L258D^ (Fig. 4*F*). These similarities in the Mg^2+^ requirement for FPP and nucleotide binding suggested that FPP could adopt a nucleotide-like binding mode in the active site of FicD.

### Structural Analysis Reveals FPP Binding to the Active Site of FicD.

**Fig. 5.**
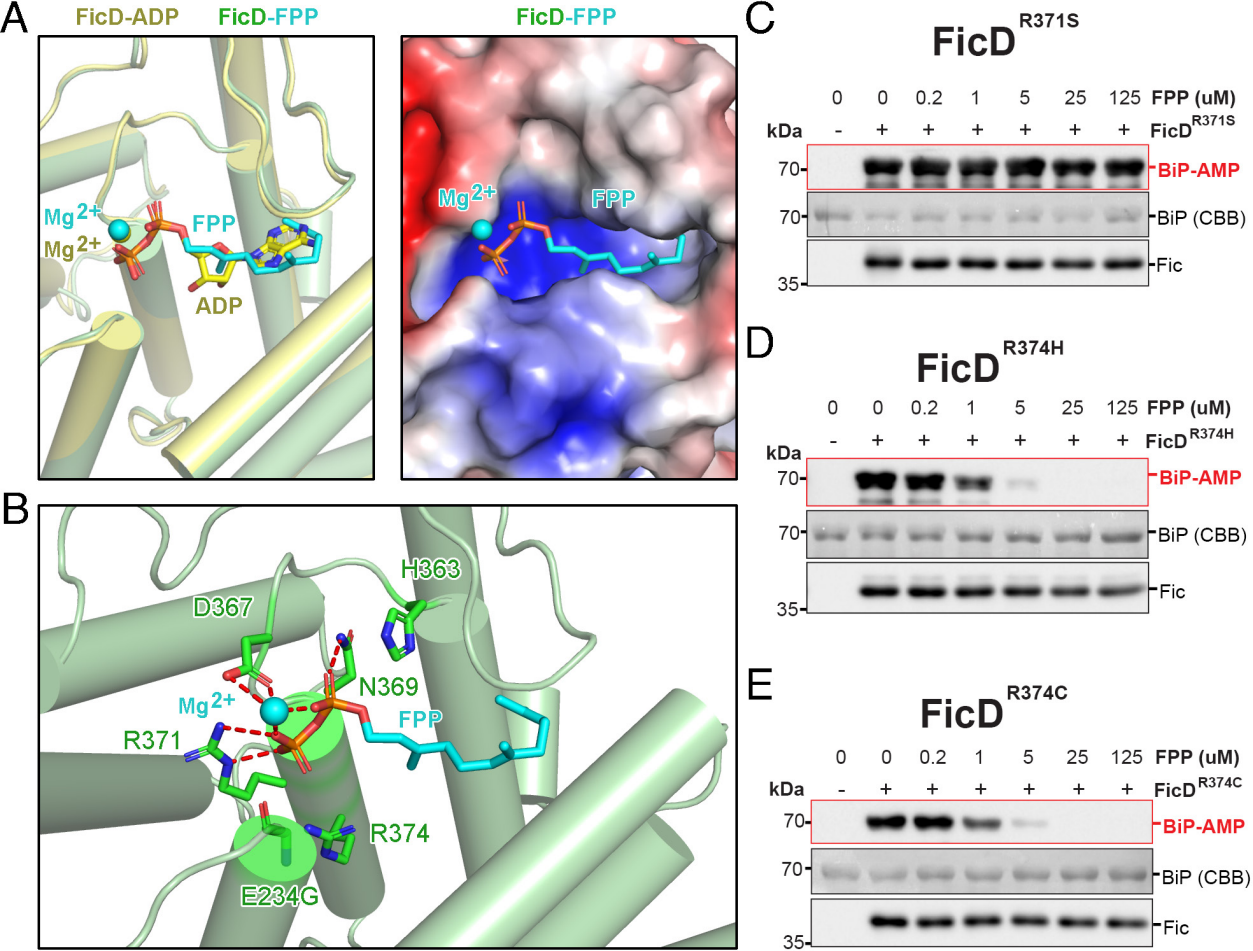
Crystal structure of FPP inhibitor bound to FicD active site. (*A*) FicD^E234G^-FPP structure in green aligned with FicD^E234G^-ADP in yellow (PDB: 4U0S) shows FPP (cyan stick) and Mg^2+^ (cyan sphere) binding the active site like Mg^2+^ADP (yellow sphere and stick) (*Left*). FPP (cyan stick) and Mg^2+^ (cyan sphere) in the pocket of FicD shown as APBS electrostatic surface with positive areas in blue, negative in red, and neutral in white (contour level: ±5 *k*_B_T/e) (*Right*). (*B*) Mg^2+^FPP (cyan) forms hydrogen bonds (dashed red lines) with the side chains of D367, N369, and R371 near the catalytic H363, but does not interact with R374. (*C*–*E*) AMPylation assays of Δ26hBiP^T229A^ in the presence of the indicated concentration of FPP using the pathogenic human variants (*C*) FicD^R371S^, (*D*) FicD^R374H^ and (*E*) FicD^R374C^. AMPylated BiP and Fic were quantified by immunodetection, and total BiP was quantified by CBB.

To reveal the mechanism of FicD inhibition by FPP, we leveraged the FicD^E234G^ mutant for structural studies. We soaked FPP into crystals of the FicD^E234G^ variant and solved the ligand-bound structure (Fig. 5 *A* and *B*). The 2.6 Å resolution ligand bound structure revealed binding of FPP to FicD active site. The diphosphate group of FPP closely overlapped with the ADP diphosphate when superimposed with a FicD^E234G^-ADP structure (Fig. 5 *A*, *Left*), and the hydrophobic isoprenyl groups of FPP overlap with the nucleoside in the active site binding pocket (Fig. 5 *A*, *Right*). The FPP diphosphate binds directly to the side chains of N369, R371, with the sidechain of D367 coordinating the Mg^2+^ (Fig. 5*B*). These interactions mimic those observed between ADP/ATP and FicD (26). Total mass analysis of FicD^WT^ or FicD^E234G^ incubated with FPP demonstrated no mass shift associated with a putative FPP automodification (*SI Appendix*, Fig. S6), consistent with noncatalytic positioning of the FPP diphosphates. As previously noted, only GPP and FPP could bind to and inhibit FicD, but analogs of the isoprenoid-pyrophosphate metabolites (geraniol, GMP, farnesol, FMP, DMAPP, and MVAPP) could not (Fig. 2 *B*–*D*). Together, these observations indicate both the pyrophosphate head and the long isoprenoid tail of GPP/FPP contribute to the binding with FicD. Importantly, this also implies potential for the FicD pocket to be targeted when designing diverse inhibitor molecules that use diphosphate as a polar head.

### Interaction of Farnesyl-Pyrophosphate with Pathogenic Variants of FicD.

Three FicD variants have been implicated in human disease. All three variants, FicD^R371S^, FicD^R374H^, and FicD^R374C^, occur at conserved active site residues. Their mutation confers total loss of deAMPylation activity while also lowering AMPylation activity (39–42). The sidechain of FicD R371 directly facilitates binding of the β-phosphates of FPP and ADP (Fig. 5*B*). Given this interaction, we predicted R371 to be critical for facilitating FPP binding and its inhibition of FicD activity. Indeed, the FicD^R371S^ variant exhibited a total loss of FPP inhibition of AMPylation activity (Fig. 5*C*). Additionally, the addition of 5 mM MgCl_2_ binding did not confer FPP binding to the FicD^R371S^ variant at any concentration tested (*SI Appendix*, Fig. S7 *A* and *B*). These results confirm that loss of FPP-mediated AMPylation inhibition was due to loss of FPP binding conferred by the FicD^R371S^ mutation.

Unlike the R371 sidechain, FicD R374 does not directly interact with FPP (Fig. 5*B*). Instead, R374 either coordinates the gamma phosphate of ATP in the AMPylation-competent state, or it forms the autoinhibitory salt bridge with E234 in the deAMPylation competent state of the enzyme (51). Therefore, mutation of R374 to His or Cys should break the autoinhibitory salt bridge, mirroring the E234G mutation that confers constitutive AMPylation. The R374H and R374C variants were not predicted to disrupt FPP binding because the gamma phosphate that R374 interacts with is missing in FPP. Indeed, using DSF, we observe shifts in the melting curves of the R374H and R374C variants upon addition of FPP. Interestingly, FPP caused shifts in the melting curves of the R374H and R374C mutants in the absence of Mg^2+^ (*SI Appendix*, Fig. S7 *C* and *D*), though the addition of Mg^2+^ enhanced the binding (*SI Appendix*, Fig. S7 *E* and *F*). These observations are consistent with R374 being required for the autoinhibitory salt bridge. In activity assays, we observe FPP inhibition of FicD^R374H^ and FicD^R374C^ mediated AMPylation (Fig. 5 *D* and *E*). Together, these data support our structural findings that FPP engages conserved active site residues analogous to ADP and highlights active site differences in the pathogenic variants that can be targeted for different inhibition profiles.

## Discussion

Metazoan FicD utilizes a bifunctional Fido domain to catalyze AMPylation and deAMPylation of the essential HSP70 chaperone BiP (23, 24). The bifunctional activity of FicD promotes adaptive engagement of UPR stress signaling by finely tuning ER chaperone capacity (21). While it is known that FicD’s bifunctional activity is influenced by ER stress, the in-vivo signaling inputs that regulate FicD activity remain unclear. Therefore, we utilized unbiased high-throughput screening (MIDAS) to identify small molecule metabolites that might regulate FicD.

Our MIDAS screen reinforced the known FicD’s specificity for binding adenine nucleotides. The thyroid hormone metabolites 3,3’-diiodothyronine and 3’-monoiodo-L-thyronine were also significantly enriched in the MIDAS screen (Fig. 1*C* and *SI Appendix*, Tables S2–S5). This observation mirrors the results of a previous drug screen with FicD that identified liothyronine as a FicD inhibitor.

Using MIDAS, we identified GPP/FPP as new PMIs for FicD, and these metabolites inhibited both AMPylation and deAMPylation activities. It is possible that independent bifunctional activities of FicD are not regulated by a small molecule metabolite but are instead regulated by another unknown cellular factor or modification, similar to other AMPylators (52). For example, FicD activity may be indirectly dictated by alterations to BiP chaperone cycling. FicD specifically recognizes the ATP-bound form of BiP (22, 53). BiP’s nucleotide binding is dynamic and regulated by several factors, such as ER Ca^2+^ levels, rate of ER ATP/ADP exchange, and action of nucleotide exchange factors, which are all affected by ER stress. As such, the possibility that FicD activity is correlated to substrate abundance has been proposed (31). As FicD is an ER membrane protein, its oligomerization activity switch may also be influenced by the membrane bilayer.

Given the localization of metazoan FicD active site to the ER lumen, the biological relevance of FPP/GPP binding and inhibition of BiP AMPylation and deAMPylation is not apparent. No known GPP/FPP utilizing enzymes localize to the luminal side of the ER membrane. Instead, the GPP/FPP inhibitors are synthesized on the cytosolic face of the ER, where they are not compatible with binding to the FicD active site. However, disregulation of lipid synthesis and UPR are known to play roles in type 2 diabetes, liver disease, and neurodegenerative disorders (54). Therefore it is tempting to speculate that unknown roles for GPP/FPP exist in th ER lumen and, therefore, could affect FicD activity. The UPR and mevalonate pathways are intrinsically linked through cholesterol and lipid synthesis, thus regulation of FicD activity by GPP/FPP could be an evolved regulatory mechanism.

Specifically, ER stress induces calcium release from the ER into the cytoplasm, which increases import of ATP into the ER via AXER (55). As ER calcium selectively increases the affinity of ADP for BiP (56), the release of calcium from the ER increases concentrations of ATP-bound BiP, the preferred substrate of FicD-mediated AMPylation. It is difficult to rationalize why BiP is constitutively deAMPlated under ER stress. However, ER stress also induces SREBP1/2-mediated expression of FPPS and other enzymes in the mevalonate pathway (57–59). Therefore, the FicD–FPP interaction could act as a temporary inhibitor for AMPylation, preventing BiP AMPylation in a calcium replete, ATP rich environment. Yet, FicD-mediated deAMPylation of AMPylated BiP would also be compromised.

As observed in the crystal structures, the diphosphates of ADP/ATP and FPP compete for engagement with conserved catalytic FicD residues (Fig. 5 *A*, *Left* and Fig. 5*B*). Given that FPP is a “pure” competitive inhibitor, its effects are overcome by high substrate concentrations. In our in vitro activity assays, substrate exceeds FPP by two to three orders of magnitude, resulting in apparent EC50s of inhibition in the low micromolar range (Fig. 2 *E* and *F*, *Right*). In the ER lumen, ATP concentrations are estimated to be 0.5 mM to 2 mM (55, 57). Total cellular concentrations of FPP have been estimated to be ~0.06 μM, which increases to ~15 μM upon blockade of squalene synthase (56). However, FPP synthesis tethered to the ER membrane via peroxisome:ER contacts could increase the local FPP concentration on the cytosolic side of the ER membrane (58–60).

Another regulatory possibility for the FPP/GPP isoprenoids could be linked to their role in glycosylation. Isoprenoids serve as precursors for a longer dolichol phosphate lipid carrier essential for N-linked protein glycosylation in the ER lumen (48). After N-linked glycosylation of ER proteins, the dolichol is released in its diphosphate form (Dol-PP) on the lumenal side of the ER membrane (52). As shown in this study, the length of isoprene tail affects the ligand–FicD interaction. The pocket of FicD in our crystal structure can accommodate FPP with 3 isoprene units (Fig. 5*A*). However, the tail of GeranylGeranyl-pyrophosphate or dolichol-pyrophosphate (with 4 or more isoprene repeats) is larger than the FicD pocket. Therefore, these molecules are not predicted to bind FicD absent a conformational change that further opens the pocket. While it is tempting to speculate that FicD activity should respond to N-linked glycosylation of newly synthesized ER proteins, whether the hydrophobic Dol-PP could partition between the membrane and the FicD active site remains to be determined. Biochemically testing this question is challenging due to the insolubility of Dol-PP.

The K_d_ of FPP binding to FicD was 60 nM. This relatively tight binding is over an order of magnitude stronger than the K_d_ for ADP (26). Despite this notable inhibition of the human FicD enzyme, the activities of two constitutively active bacterial Fido proteins, AvrB and VopS, were not inhibited by FPP (*SI Appendix*, Fig. S5). However, the Fido domains of AvrB and VopS are structurally and functionally distinct from FicD (20, 25, 30). Neither are intramolecularly autoinhibited nor are they bifunctional. Indeed, it is possible that GPP/FPP binding requires autoinhibition and that other autoinhibited Fido proteins interact with GPP/FPP. Approximately 90% of all AMPylating Fido domains are predicted to be intrinsically inhibited and bifunctional, a majority of which are in bacteria (32). Like metazoan FicD, these enzymes play a housekeeping role, modifying DNA gyrases and topoisomerases to tune bacterial stress signaling (61, 62). The bacterial Fido domain proteins are localized to the cytoplasm where terpenoid biosynthesis from FPP intermediates is performed (63, 64). Therefore, our findings may simply reflect an evolutionary link that remains between bacterial housekeeping Fido domains that function in stress signaling and their metazoan counterparts that retain intramolecular autoinhibition.

FicD^R371S^, FicD^R374H^, and FicD^R374C^ variants are implicated in human disease. FicD^R371S^ causes a rare form of neonatal diabetes with severe neurodevelopmental impairment (39). FicD^R374H^ and FicD^R374C^ are implicated in a progressive motor neuron disease and increased risk of diabetes in adulthood (41, 42). In all three variants, loss of BiP deAMPylation is central to the mechanism of disease (39, 41, 42). FicD^R374H^ and FicD^R374C^ retain interaction with FPP and their AMPylation activity is inhibited by the metabolite (Fig. 5 *D* and *E* and *SI Appendix*, Fig. S7 *C–F*). Conversely, AMPylation activity of the FicD^R371S^ variant lacking FPP binding was not inhibited (Fig. 5*C* and *SI Appendix*, Fig. S7 *A* and *B*). These results are consistent with the contribution of R371 and R374 to FPP binding observed in our crystal structure (Fig. 5*B*).

A preclinical FicD^R371S^ mouse model demonstrated progressive loss of pancreatic islet organization with defects in insulin secretion (40), which mirrors phenotypes in FicD^R371S^ homozygote children (39). In the FicD^R374H^ and FicD^R374C^ variants, loss of intramolecular autoinhibition confers loss of deAMPylation activity. This mechanism mirrors the biochemically well-characterized FicD^E234G^, which is homozygous lethal in both flies (24) and mice (38, 43). The E234G mutation confers enhanced Mg^2+^ independent binding of FPP, which was also observed in the FicD^R374H^ and FicD^R374C^ mutants. Therefore, our crystal structure of FPP bound to FicD may provide an experimentally grounded foundation for future medicinal chemistry efforts to develop inhibitors to treat pathologic FicD AMPylation caused by the FicD^R374H^ and FicD^R374C^ mutations. We have observed that the *FicD* null mice show mild phenotypes when stressed or aged but are otherwise viable. Therefore, inhibiting the AMPylation of FicD^R374H^ or FicD^R374C^ should mimic a FicD null and might alleviate early phenotypes observed with the constitutive AMPylation. In addition, over time, the inhibition of both FicD AMPylation and deAMPylation would likely resolve toward no observable BiP AMPylation due to turnover of endogenous BiP.

In summary, we characterize the interactions of FPP with FicD in precise biochemical and structural details. The FPP specificity and its potency of action in inhibiting FicD is particularly striking. FPP binds to the FicD active site with over an order of magnitude higher affinity than ADP. The observed interactome of FicD with natural ligands was also markedly small due to our triage strategy. These included nucleotides, thyroid hormone intermediates, and GPP/FPP. In addition, the interaction with GPP/FPP may be exclusive to intramolecularly autoinhibited Fic proteins. Together, these observations implicate a physiological role for isoprenoid metabolites and FicD activity. Finally, we define dADP and dATP as FicD ligands, potentially expanding the repertoire of cosubstrates utilized by the Fido domain. Together, our work provides novel insight into ligands that can bind to the active site of the FicD, while revealing catalytic specificity for human disease variants.

## Materials and Methods

Detailed descriptions of the experimental methods are provided in *SI Appendix*. These include reagents, high-throughput screening, data analysis of screening data, cloning of genes, protein purification, in vitro activity and thermal shift assays, protein crystallography, and isothermal titration calorimetry.

## Supplementary Material

Appendix 01 (PDF)

Dataset S01 (XLSX)

Dataset S02 (XLSX)

Dataset S03 (XLSX)

Dataset S04 (XLSX)

## Data Availability

The atomic coordinates of the corresponding crystal structure are deposited in the Protein Data Bank with accession code 9YZ5 (extended ID pdb_00009YZ5) (65). All other data are included in the manuscript and/or supporting information.

## References

[1] L. M. Hendershot, T. M. Buck, J. L. Brodsky, The essential functions of molecular chaperones and folding enzymes in maintaining endoplasmic reticulum homeostasis. J. Mol. Biol. **436**, 168418 (2024). 38143019 10.1016/j.jmb.2023.168418 PMC12015986

[2] I. Braakman, D. N. Hebert, Protein folding in the endoplasmic reticulum. Cold Spring Harb. Perspect. Biol. **5**, a013201 (2013). 23637286 10.1101/cshperspect.a013201 PMC3632058

[3] V. Gonzalez-Teuber , Small molecules to improve ER proteostasis in disease. Trends Pharmacol. Sci. **40**, 684–695 (2019). 31377018 10.1016/j.tips.2019.07.003

[4] X. Chen, C. Shi, M. He, S. Xiong, X. Xia, Endoplasmic reticulum stress: Molecular mechanism and therapeutic targets. Signal Transduct. Target. Ther. **8**, 352 (2023). 37709773 10.1038/s41392-023-01570-w PMC10502142

[5] C. Hetz, K. Zhang, R. J. Kaufman, Mechanisms, regulation and functions of the unfolded protein response. Nat. Rev. Mol. Cell Biol. **21**, 421–438 (2020). 32457508 10.1038/s41580-020-0250-z PMC8867924

[6] C. Lebeaupin, J. Yong, R. J. Kaufman, The impact of the ER unfolded protein response on cancer initiation and progression: Therapeutic implications. Adv. Exp. Med. Biol. **1243**, 113–131 (2020). 32297215 10.1007/978-3-030-40204-4_8 PMC7243802

[7] B. M. Gardner, D. Pincus, K. Gotthardt, C. M. Gallagher, P. Walter, Endoplasmic reticulum stress sensing in the unfolded protein response. Cold Spring Harb. Perspect. Biol. **5**, a013169 (2013). 23388626 10.1101/cshperspect.a013169 PMC3578356

[8] D. Pincus , BiP binding to the ER-stress sensor Ire1 tunes the homeostatic behavior of the unfolded protein response. PLoS Biol. **8**, e1000415 (2010). 20625545 10.1371/journal.pbio.1000415 PMC2897766

[9] A. Bertolotti, Y. Zhang, L. M. Hendershot, H. P. Harding, D. Ron, Dynamic interaction of BiP and ER stress transducers in the unfolded-protein response. Nat. Cell Biol. **2**, 326–332 (2000). 10854322 10.1038/35014014

[10] J. Shen, X. Chen, L. Hendershot, R. Prywes, ER stress regulation of ATF6 localization by dissociation of BiP/GRP78 binding and unmasking of Golgi localization signals. Dev. Cell **3**, 99–111 (2002). 12110171 10.1016/s1534-5807(02)00203-4

[11] A. Bakunts , Ratiometric sensing of BiP-client versus BiP levels by the unfolded protein response determines its signaling amplitude. Elife **6**, e27518 (2017). 29251598 10.7554/eLife.27518 PMC5792092

[12] M. Vitale , Inadequate BiP availability defines endoplasmic reticulum stress. Elife **8**, e41168 (2019). 30869076 10.7554/eLife.41168 PMC6417858

[13] D. Acosta-Alvear, J. M. Harnoss, P. Walter, A. Ashkenazi, Homeostasis control in health and disease by the unfolded protein response. Nat. Rev. Mol. Cell Biol. **26**, 193–212 (2025). 39501044 10.1038/s41580-024-00794-0

[14] L. Plate, R. L. Wiseman, Regulating secretory proteostasis through the unfolded protein response: From function to therapy. Trends Cell Biol. **27**, 722–737 (2017). 28647092 10.1016/j.tcb.2017.05.006 PMC5612838

[15] S. J. Marciniak, J. E. Chambers, D. Ron, Pharmacological targeting of endoplasmic reticulum stress in disease. Nat. Rev. Drug Discov. **21**, 115–140 (2022). 34702991 10.1038/s41573-021-00320-3

[16] C. Hetz, J. M. Axten, J. B. Patterson, Pharmacological targeting of the unfolded protein response for disease intervention. Nat. Chem. Biol. **15**, 764–775 (2019). 31320759 10.1038/s41589-019-0326-2

[17] H. Ham , Unfolded protein response-regulated Drosophila Fic (dFic) protein reversibly AMPylates BiP chaperone during endoplasmic reticulum homeostasis. J. Biol. Chem. **289**, 36059–36069 (2014). 25395623 10.1074/jbc.M114.612515 PMC4276871

[18] A. Sanyal , A novel link between Fic (filamentation induced by cAMP)-mediated adenylylation/AMPylation and the unfolded protein response. J. Biol. Chem. **290**, 8482–8499 (2015). 25601083 10.1074/jbc.M114.618348 PMC4375499

[19] S. Preissler , AMPylation matches BiP activity to client protein load in the endoplasmic reticulum. Elife **4**, e12621 (2015). 26673894 10.7554/eLife.12621 PMC4739761

[20] L. N. Kinch, M. L. Yarbrough, K. Orth, N. V. Grishin, Fido, a novel AMPylation domain common to fic, doc, and AvrB. PLoS One **4**, e5818 (2009). 19503829 10.1371/journal.pone.0005818 PMC2686095

[21] A. K. Casey , Fic-mediated AMPylation tempers the unfolded protein response during physiological stress. Proc. Natl. Acad. Sci. U.S.A. **119**, e2208317119 (2022). 35914137 10.1073/pnas.2208317119 PMC9371680

[22] S. Preissler , AMPylation targets the rate-limiting step of BiP’s ATPase cycle for its functional inactivation. Elife **6**, e29428 (2017). 29064368 10.7554/eLife.29428 PMC5667935

[23] S. Preissler, C. Rato, L. Perera, V. Saudek, D. Ron, FICD acts bifunctionally to AMPylate and de-AMPylate the endoplasmic reticulum chaperone BiP. Nat. Struct. Mol. Biol. **24**, 23–29 (2017). 27918543 10.1038/nsmb.3337 PMC5221731

[24] A. K. Casey , Fic-mediated deAMPylation is not dependent on homodimerization and rescues toxic AMPylation in flies. J. Biol. Chem. **292**, 21193–21204 (2017). 29089387 10.1074/jbc.M117.799296 PMC5743091

[25] M. L. Yarbrough , AMPylation of Rho GTPases by Vibrio VopS disrupts effector binding and downstream signaling. Science **323**, 269–272 (2009). 19039103 10.1126/science.1166382

[26] T. D. Bunney , Crystal structure of the human, FIC-domain containing protein HYPE and implications for its functions. Structure **22**, 1831–1843 (2014). 25435325 10.1016/j.str.2014.10.007 PMC4342408

[27] B. Gulen, A. Itzen, Revisiting AMPylation through the lens of Fic enzymes. Trends Microbiol. **30**, 350–363 (2022). 34531089 10.1016/j.tim.2021.08.003

[28] P. Engel , Adenylylation control by intra- or intermolecular active-site obstruction in Fic proteins. Nature **482**, 107–110 (2012). 22266942 10.1038/nature10729

[29] A. Goepfert, F. V. Stanger, C. Dehio, T. Schirmer, Conserved inhibitory mechanism and competent ATP binding mode for adenylyltransferases with Fic fold. PLoS One **8**, e64901 (2013). 23738009 10.1371/journal.pone.0064901 PMC3667792

[30] W. Peng , Pseudomonas effector AvrB is a glycosyltransferase that rhamnosylates plant guardee protein RIN4. Sci. Adv. **10**, eadd5108 (2024). 38354245 10.1126/sciadv.add5108 PMC10866546

[31] L. A. Perera , An oligomeric state-dependent switch in the ER enzyme FICD regulates AMPylation and deAMPylation of BiP. EMBO J. **38**, e102177 (2019). 31531998 10.15252/embj.2019102177 PMC6826200

[32] F. V. Stanger , Intrinsic regulation of FIC-domain AMP-transferases by oligomerization and automodification. Proc. Natl. Acad. Sci. U.S.A. **113**, E529–E537 (2016). 26787847 10.1073/pnas.1516930113 PMC4747691

[33] S. Veyron , A Ca(2+)-regulated deAMPylation switch in human and bacterial FIC proteins. Nat. Commun. **10**, 1142 (2019). 30850593 10.1038/s41467-019-09023-1 PMC6408439

[34] M. Rahman , Visual neurotransmission in Drosophila requires expression of Fic in glial capitate projections. Nat. Neurosci. **15**, 871–875 (2012). 22544313 10.1038/nn.3102 PMC3578554

[35] A. G. Lobato , Loss of Fic causes progressive neurodegeneration in a Drosophila model of hereditary spastic paraplegia. Biochim. Biophys. Acta **1870**, 167348 (2024). 10.1016/j.bbadis.2024.167348 PMC11549967 38986817

[36] A. K. Casey , Ficd regulates adaptation to the unfolded protein response in the murine liver. Biochimie **225**, 114–124 (2024). 38740171 10.1016/j.biochi.2024.05.012 PMC12163935

[37] S. M. Lacy , FICD (FIC domain protein adenylyl transferase) deficiency protects mice from hypertrophy-induced heart failure and promotes BiP (binding immunoglobulin protein) -mediated activation of the unfolded protein response and endoplasmic reticulum-selective autophagy in cardiomyocytes. J. Am. Heart Assoc. **14**, e040192 (2025). 40820977 10.1161/JAHA.124.040192 PMC12748080

[38] K. M. Van Pelt, M. C. Truttmann, Loss of FIC-1-mediated AMPylation activates the UPRER and upregulates cytosolic HSP70 chaperones to suppress polyglutamine toxicity. PLoS Genet. **21**, e1011723 (2025). 40512832 10.1371/journal.pgen.1011723 PMC12193957

[39] L. A. Perera , Infancy-onset diabetes caused by de-regulated AMPylation of the human endoplasmic reticulum chaperone BiP. EMBO Mol. Med. **15**, e16491 (2023). 36704923 10.15252/emmm.202216491 PMC9994480

[40] A. K. Casey , Pre-clinical model of dysregulated FicD AMPylation causes diabetes by disrupting pancreatic endocrine homeostasis. Mol. Metab. **95**, 102120 (2025). 40073934 10.1016/j.molmet.2025.102120 PMC11964657

[41] A. P. Rebelo , BiP inactivation due to loss of the deAMPylation function of FICD causes a motor neuron disease. Genet. Med. **24**, 2487–2500 (2022). 36136088 10.1016/j.gim.2022.08.019

[42] A. P. Rebelo , FIC domain protein adenylyltransferase (FICD)-related complex hereditary spastic paraplegia with diabetes mellitus. Mov. Disord. Clin. Pract. **12**, 1193–1195 (2025). 40062579 10.1002/mdc3.70033 PMC12371442

[43] N. McCaul , Deletion of mFICD AMPylase alters cytokine secretion and affects visual short-term learning in vivo. J. Biol. Chem. **297**, 100991 (2021). 34419450 10.1016/j.jbc.2021.100991 PMC8441161

[44] K. G. Hicks , Protein-metabolite interactomics of carbohydrate metabolism reveal regulation of lactate dehydrogenase. Science **379**, 996–1003 (2023). 36893255 10.1126/science.abm3452 PMC10262665

[45] B. K. Chatterjee , Small-molecule FICD inhibitors suppress endogenous and pathologic FICD-mediated protein AMPylation. ACS Chem. Biol. **20**, 880–895 (2025). 40036289 10.1021/acschembio.4c00847 PMC12007993

[46] A. B. Uceda, L. Mariño, R. Casasnovas, M. Adrover, An overview on glycation: Molecular mechanisms, impact on proteins, pathogenesis, and inhibition. Biophys. Rev. **16**, 189–218 (2024). 38737201 10.1007/s12551-024-01188-4 PMC11078917

[47] M. Kanehisa, Y. Sato, M. Kawashima, M. Furumichi, M. Tanabe, KEGG as a reference resource for gene and protein annotation. Nucleic Acids Res. **44**, D457–D462 (2015). 26476454 10.1093/nar/gkv1070 PMC4702792

[48] M. B. Jones, J. N. Rosenberg, M. J. Betenbaugh, S. S. Krag, Structure and synthesis of polyisoprenoids used in N-glycosylation across the three domains of life. Biochim. Biophys. Acta **1790**, 485–494 (2009). 19348869 10.1016/j.bbagen.2009.03.030 PMC2755495

[49] K. F. R. Pobre, G. J. Poet, L. M. Hendershot, The endoplasmic reticulum (ER) chaperone BiP is a master regulator of ER functions: Getting by with a little help from ERdj friends. J. Biol. Chem. **294**, 2098–2108 (2019). 30563838 10.1074/jbc.REV118.002804 PMC6369273

[50] J. Wei, J. R. Gaut, L. M. Hendershot, In vitro dissociation of BiP-peptide complexes requires a conformational change in BiP after ATP binding but does not require ATP hydrolysis. J. Biol. Chem. **270**, 26677–26682 (1995). 7592894 10.1074/jbc.270.44.26677

[51] L. A. Perera , Structures of a deAMPylation complex rationalise the switch between antagonistic catalytic activities of FICD. Nat. Commun. **12**, 5004 (2021). 34408154 10.1038/s41467-021-25076-7 PMC8373988

[52] A. K. Casey, K. Orth, Enzymes involved in AMPylation and deAMPylation. Chem. Rev. **118**, 1199–1215 (2018). 28819965 10.1021/acs.chemrev.7b00145 PMC5896785

[53] J. Fauser , Specificity of AMPylation of the human chaperone BiP is mediated by TPR motifs of FICD. Nat. Commun. **12**, 2426 (2021). 33893288 10.1038/s41467-021-22596-0 PMC8065156

[54] W. Bialek , The lipid side of unfolded protein response. Biochim. Biophys. Acta Mol. Cell Biol. Lipids **1869**, 159515 (2024). 38844203 10.1016/j.bbalip.2024.159515

[55] J. Yong , Mitochondria supply ATP to the ER through a mechanism antagonized by cytosolic Ca(2). Elife **8**, e49682 (2019). 31498082 10.7554/eLife.49682 PMC6763289

[56] H. Tong, S. A. Holstein, R. J. Hohl, Simultaneous determination of farnesyl and geranylgeranyl pyrophosphate levels in cultured cells. Anal. Biochem. **336**, 51–59 (2005). 15582558 10.1016/j.ab.2004.09.024

[57] N. Vishnu , ATP increases within the lumen of the endoplasmic reticulum upon intracellular Ca2+ release. Mol. Biol. Cell **25**, 368–379 (2014). 24307679 10.1091/mbc.E13-07-0433 PMC3907277

[58] L. M. Olivier, W. Kovacs, K. Masuda, G. A. Keller, S. K. Krisans, Identification of peroxisomal targeting signals in cholesterol biosynthetic enzymes. AA-CoA thiolase, hmg-coa synthase, MPPD, and FPP synthase. J. Lipid Res. **41**, 1921–1935 (2000). 11108725

[59] S. K. Krisans, J. Ericsson, P. A. Edwards, G. A. Keller, Farnesyl-diphosphate synthase is localized in peroxisomes. J. Biol. Chem. **269**, 14165–14169 (1994). 8188698

[60] M. Schuldiner, E. Zalckvar, Incredibly close-a newly identified peroxisome-ER contact site in humans. J. Cell Biol. **216**, 287–289 (2017). 28108527 10.1083/jcb.201701072 PMC5294797

[61] A. Harms , Adenylylation of gyrase and Topo IV by FicT toxins disrupts bacterial DNA topology. Cell Rep. **12**, 1497–1507 (2015). 26299961 10.1016/j.celrep.2015.07.056

[62] C. Lu, E. S. Nakayasu, L. Q. Zhang, Z. Q. Luo, Identification of Fic-1 as an enzyme that inhibits bacterial DNA replication by AMPylating GyrB, promoting filament formation. Sci. Signal. **9**, ra11 (2016). 26814232 10.1126/scisignal.aad0446

[63] E. J. N. Helfrich, G. M. Lin, C. A. Voigt, J. Clardy, Bacterial terpene biosynthesis: Challenges and opportunities for pathway engineering. Beilstein J. Org. Chem. **15**, 2889–2906 (2019). 31839835 10.3762/bjoc.15.283 PMC6902898

[64] J. Pérez-Gil, M. Rodríguez-Concepción, Metabolic plasticity for isoprenoid biosynthesis in bacteria. Biochem. J. **452**, 19–25 (2013). 23614721 10.1042/BJ20121899

[65] W. Peng, K. Orth, Human FicD bound with farnesyl pyrophosphate. Protein Data Bank. https://www.rcsb.org/structure/9YZ5. Deposited 30 October 2025.

